# Anti-hyperglycaemic activity of *Moringa oleifera* is partly mediated by carbohydrase inhibition and glucose-fibre binding

**DOI:** 10.1042/BSR20170059

**Published:** 2017-06-30

**Authors:** Sohel Bin Azad, Prawej Ansari, Shofiul Azam, Saad Mosharraf Hossain, Mohammad Ibtida-Bin Shahid, Mahmudul Hasan, J.M.A. Hannan

**Affiliations:** Department of Pharmaceutical Sciences, School of Health and Life Sciences, North South University, Dhaka 1229, Bangladesh

**Keywords:** glucose tolerance, ELISA, hypoglycemia, type 2 diabetes

## Abstract

*Moringa oleifera* has potential anti-hyperglycaemic effects that have been reported earlier by different scientific groups using animal models of diabetes. We aimed to explore the possible mechanisms of action of *M. oleifera* extract through different methods. Primarily, we measured fasting blood glucose and performed glucose tolerance test, in Type 2 diabetic rats. Further, we studied the effects of extracts on pancreatic insulin concentration. Extracts’ effect on carbohydrate breakdown was assayed using α-amylase inhibition assays and assay of six different segments of gastrointestinal (GI) tracts. An *in situ* intestinal perfusion model and a glucose fibre assay were performed to see the potentiality of *M. oleifera* on glucose absorption. * M. oleifera* showed no significant change in insulin secretion *in vivo*. Additionally, substantial effect of the extract was seen on retarded glucose absorption and in the *in situ* perfusion study of rat intestinal model. α-amylase action was inhibited by the extract, yet again, these findings were further confirmed via the Six Segment assay, where sucrose digestion was found to be inhibited throughout the length of the GI tract. A combined *in vitro*, *in vivo* and *in situ* tests justified the potential of anti-hyperglycaemic activity of *M. oleifera* and its tissue level mechanism is also justified.

## Introduction

For the last few decades, diabetes has held its position as one of the world’s predominant endocrine disorder [1]. By nature, it cannot be completely cured but it should be kept under tight control. Usually modified lifestyle, medications, diet or a combination of all these are prescribed to diabetic patients to control diabetes. Complementary and alternative medicines (CAMs) have become popular among people from developing countries for managing a multitude of disorders including diabetes. However, unclear mechanism of action and lack of scientific evidence of efficacy of these therapies keeps them far behind from important use. Another drawback of these plants used in various ailments is that they have no established safety profile; although it is thought that they are safe besides being economical, effective and their easy availability. Traditional practitioners globally emphasize these advantages of medicinal plants in their day-to-day practice. Traditional use of medicines is recognized as a way to learn about potential future medicines. In 2001, researchers identified 122 compounds used in mainstream medicine that were derived from ‘ethnomedical’ plant sources; 80% of these compounds were used in the same or related manner as the traditional ethnomedical use [2].

*Moringa oleifera* is also known as *Moringa pterygosperma* Gaertn, is a member of the Moringaceae family of perennial angiosperm plants, which includes 12 other species. This plant is commonly named in English as *Moringa* and drumstick tree [3]. *M. oleifera* is one of the most useful tropical trees [4], it has the following characteristics: high protein, vitamins, minerals and carbohydrate content. This plant provides high value of nutrition for both humans and domestic animals; the seeds contain high oil content (42%), which is edible and has medicinal uses. Previous studies by Amaglo et al. [5], Limon-Pacheco and Gonsebatt [6] and Mahajan and Mehta [7] have reported different pharmacological potentialities including antioxidant and anti-inflammatory properties of *M. oleifera*. Moreover, Awodele et al. [8] have studied to evaluate toxicological property of the aqueous extract of *M. oleifera* Lam. (Moringaceae). Oyedepo et al. [9] evaluated the anti-hyperlipidaemic effect of aqueous leaves extract of *M. oleifera* while Gupta et al. [10] worked on the evaluation of anti-diabetic and antioxidant activity of *M. oleifera* in experimental diabetes model. Again, Jaiswal et al. [11] investigated the role of *M. oleifera* in regulation of diabetes-induced oxidative stress, while Choudhary et al. [12] assessed the anti-ulcer potential of *M. oleifera* root bark extract in rats. Although there have been several reports on the cholesterol and blood glucose reducing effect of different fractions of leaf extracts of *M. oleifera* in rats [13–17], there is still paucity of information on the hypoglycaemic activity of the extract at the doses investigated in the present study.

However, basic mechanism of action of *M. oleifera* still remains unclear till today as an anti-hyperglycaemic herb. The aim of the present study is to paint a comprehensive picture of effects of *M. oleifera* on sucrose breakdown and glucose absorption, insulin release and intestinal enzyme functions. Our study will help to identify the particular organ or organ system responsible for the previously seen hypoglycaemic activity of this plant.

## Materials and methods

### Plant collection and processing

*M. oleifera* leaves were collected from Jahangirnagar University, University Ayurvedic Research Centre (UARC), Dhaka, Bangladesh. The plant was identified by a taxonomist prior to further processing and a voucher specimen was deposited at the National Herbarium at Mirpur, Dhaka, Bangladesh with accession number DACB-12290. Then, the leaves were cleaned off of dirt and other debris and thoroughly washed under running tap water followed by air drying for granules preparation.

The solvent (ethanol 80%) was collected from organic chemistry laboratory of North South University, Dhaka. Approximately 400 g of ground powder was taken in flat-bottomed container, in which 2000 ml of solvent was added gradually with regular stirring. The container was sealed and left in the mechanical shaker for 6 days, on an average 2–3 h daily. The mixture was filtrated in stepwise processes. A clean, white cotton cloth was used which was double folded and the mixture was passed through it. It was the primary filtration where larger fibres were separated from the mixture. Then cotton was used later to filter the mixture to separate larger materials. Finally, filter paper was used for the separation of clear filtrate from the mixture. The amount of filtrate obtained was 150 ml. The filtrate (ethanol extract) obtained was evaporated by Rotary evaporator (Bibby RE-200, Sterilin Ltd., U.K.) at 5–6 rpm and at 57°C. It rendered a gummy concentrate of greenish colour that was designated as crude extract or ethanolic extract. This crude ethanolic extract was then dried by freeze drier and preserved at 4°C until further use.

### Animal handling

Long Evan type normal healthy and Type 2 diabetic rats of both sexes were farmed in the animal house of the Department of Pharmaceutical Sciences, North South University, Dhaka and the weights of rats were approximately 180–220 g. All test animals were kept at an ambient temperature of 22 ± 5°C and humidity was maintained at 50–70%. Twelve hours day-night cycle was maintained to avoid fluctuations in the circadian rhythm and the rats were kept in translucent plastic cages with wood shavings provided as bedding; while grilled cages were replaced with bedding prior to fasted rat testing, to prevent corpophagy. Standard rat pellets and filtered drinking water were provided to the test animals *ad libitum* throughout the experiment apart from certain tests that required fasting and during that time only water was given to them. The experimental protocol was designed and subsequently approved by the Ethics Committee on Animal Research, North South University, following the ‘Revised guide for the care and use of laboratory animals by American Physiological Society’ [18].

### Diabetes induction

Intraperitoneal streptozotocin (STZ) was given to normal and healthy rats to induce Type 2 diabetes in citrate buffer solution at a dose of 90 mg/kg. Newly born rats aged less than 48 h and weighing 7 g were chosen for this procedure. Three months later, fasting blood glucose levels of 8–12 mmol/l were selected for the experiments and an oral glucose tolerance test (OGTT) was performed for further confirmation of Type 2 diabetes [19].

### Acute effects of ethanolic extracts of *M. oleifera* on glucose homoeostasis

*M. oleifera* extract was suspended in distilled water and orally administered to 12-h fasted rats and control group animals received only an equal volume of distilled water; to test blood glucose change in fasting condition. Effects on glucose tolerance were similarly evaluated by administration of *M. oleifera* extracts together with glucose (2.5 g/10 ml per kg body weight) after a fasting period of 12 h and control group received only glucose solution. In either cases, blood sample was collected from the tail vein of rats, serum separated by centrifugation and stored at –22°C until further analysis. Blood glucose was analysed by GOD-PAP method [20] (Glucose kit, Randox™, U.K.).

### Effects of *M. oleifera* on plasma insulin

Blood was drawn out from Type 2 diabetic rats, 1 h after administration of *M. oleifera*. The amount of insulin released from the pancreas *in vivo*, was determined using Rat Insulin ELISA Kit (Crystal Chem™, U.S.A.).

### Effects of *M. oleifera* on intestinal glucose absorption

An *in situ* intestinal perfusion technique [21] was used to determine the effect of *M. oleifera* intestinal absorption of glucose in 36-h fasted non-diabetic rats anaesthetized using Ketamine (80 mg/kg). Ethanol extract of *M. oleifera* (10 mg/ml equivalent to 0.5 g/kg was suspended in Krebs Ringer buffer, along with glucose (54 g/l). These were passed through rat pyloris via a butterfly cannula and the perfusate was collected by means of a tube inserted at the end of ileum. The control group was perfused with Krebs Ringer buffer along with glucose only. Perfusion was carried out at a rate of 0.5 ml/min for 30 min at 37°C. The results were presented as percentage of absorbed glucose, calculated from the percentage change in the amount of glucose in solution before and after the perfusion.

### Effects of *M. oleifera* on sucrose absorption from the gut

The effect of *M. oleifera* on sucrose absorption from gastrointestinal tract (GIT) was assayed by determining the unabsorbed sucrose content following oral sucrose load by sucrose malabsorption study as described by Hannan *et al*. [22].Twelve hours fasted, Type 2 diabetic rats were administered 50% sucrose solution per oral (2.5 g/kg body mass) along with 500 mg/kg dose of *M. oleifera* and equal volume of water for control. Blood was sampled at the following time intervals: 30, 60, 120 and 240 min, after sucrose load for the quantification of blood glucose. At these time intervals, some of the rats were killed for determining unabsorbed sucrose contents of the GI tract. The GI tract was excised and separated into six segments: the stomach, the upper 20 cm, middle and lower 20 cm of the small intestine, the caecum and the large intestine. Each segment was rinsed with acidified, ice-cold, saline followed by centrifugation at 3000 rpm (1000 ***g***) for 10 min. The supernatant was pipetted out and boiled for 2 h in sulfuric acid to hydrolyse the sucrose. The sulfuric acid was later neutralized by NaOH solution. Both plasma glucose concentration and the amount of glucose released from residual sucrose in the GI tract was determined. The GI sucrose content was calculated from the amount of liberated glucose [23].

### Effects of *M. oleifera* on gut motility

GI motility was determined by means of BaSO_4_ milk following the previously described method of Chattarjee [24]. BaSO_4_ milk was prepared by mixing BaSO_4_ as 10% (w/v) in 0.5% CM-cellulose to form a suspension. The ethanol extract was administered orally, 1 h before the oral administration of BaSO_4_ milk. Control group was administered distilled water only (10 ml/kg). Rats belonging to all groups were killed 15 min after BaSO_4_ administration. The distance travelled by BaSO_4_ milk was measured and represented as a percentage of total length of the small intestine (from pylorus to ileocaecal junction).

### Effect of *M. oleifera* on jejunal nutrient absorption by glucose dialysis tube retardation assay

Dry, precut dialysis sacs (inflated diameter approximately 16 mm, length =30 cm, Sigma–Aldrich™, U.S.A.) were soaked in 1 g sodium azide/l. The bag was loaded with 6 ml sodium azide (1 gm/l) and 36 mg glucose alone (the control sac) or after addition of fine powder of *M. oleifera*. The dry fibrous powder was wetted by an aqueous solution of sodium azide (1 g/l) for 14 h prior to the experiment. The sacs were closed at the ends and hung in a solution of 100 ml of sodium azide (1 g/l) and then placed in a stirred bath at 37°C for 1 h. At 30 and 60 min time interval, 2 ml of the dialysate was analysed for glucose by the GOD-PAP method as previously described.

The effect of fibre on nutrient absorption was indicated by the glucose dialysis retardation index (GDRI):

$$-\left(\frac{\text{Total glucose diffused from sac containing fibre }\times 100}{\text{Total glucose diffused from sac containing no fibre present}}\right)$$

### Effect of *M. oleifera* on α-amylase activity

The effects of *M. oleifera* powder on starch digestibility was determined as a function of time in a fibre-enzyme-starch mixture system using a dialysis membrane with a cut-off molecular weight of 12000 Da (inflated diameter approximately 16 mm, length =30 cm, Sigma–Aldrich™, U.S.A.) as previously described with minor modifications [25 ]. A solution was prepared by mixing 0.2 g powdered *M. oleifera* and 0.04 g α-amylase (obtained from human saliva, Sigma–Aldrich™, U.S.A.) in 10 ml of potato starch solution (4 g/100 ml) was dialysed in 200 ml deionised water at 37°C. Following the incubation period: 10, 30, 60, and 120 min, glucose concentration in the dialysate solution was assayed using the GOD-PAP method as described previously. The control was run without the addition of powder.

### Determination of glucose-adsorption capacity

The assay was conducted following the procedure by Ou et al. [26], where the glucose-adsorption ability (mM/mol g^–1^) was measured by mixing 1 g of insoluble plant powder or CM-cellulose with 100 ml of glucose solution at a constant temperature of 37°C for 6 h. This was then followed by centrifugation at 3500 rpm for 15 min. Glucose concentration in the supernatant was assayed using GOD-PAP method as previously described.

### Statistical analysis

Statistical tests were conducted using GraphPad Prism 6. Results are presented as means ± S.E.M. Experiments with data being collected at several time intervals, were analysed using repeated-measures ANOVA followed by Bonferroni adjustment ensuring an error margin within ≤5%. One-way ANOVA was carried out and pairwise comparisons were made with the control group using Dunnett’s test to maintain an acceptable error margin of 5%. A two-tailed *P* value of <0.05 was considered statistically significant.

## Results

### Acute effects of *M. oleifera* on glucose homoeostasis

**Figure 1:**
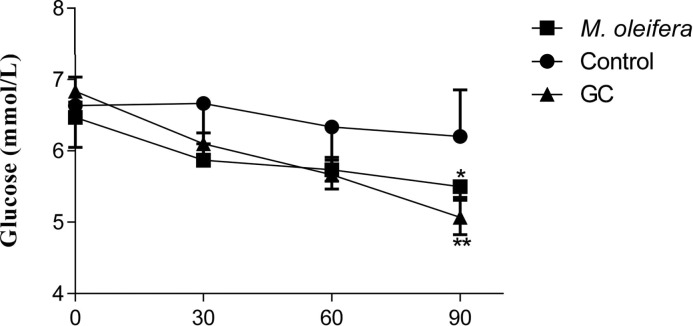
Effects of ethanol extract of M. oleifera on fasting blood glucose level in Type 2 diabetic rats Values are means ± S.E.M represented by vertical bars (*n*=10). Fasted rats were given ethanol extract of *M. oleifera* (500 mg/kg) or Glibenclamide (GC) (0.5 mg/kg) or only water (control) by oral administration. Mean values marked with an asterisk (*) were significantly different from those of respective control rats (^*^P <0.05 and ,^**^P<0.001) (derived from repeated-measures ANOVA and adjusted using Bonferroni correction).

**Figure 2:**
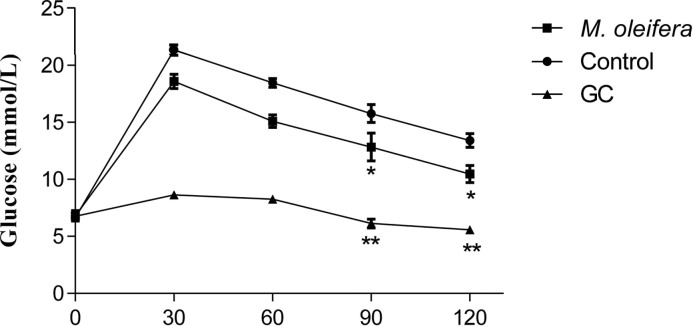
Effects of ethanol extract of *M. oleifera* on glucose tolerance in Type 2 diabetic rats Values are means ± S.E.M represented by vertical bars (*n*=10). Fasted rats were given ethanol extract of *M. oleifera* (500 mg/kg body weight) or Glibenclamide (GC) (0.5 mg/kg) or only water (control) by oral administration with glucose (2.5 g/kg body weight). Mean values marked with an asterisk (*) were significantly different from those of respective control rats (^*^P <0.05, **P<0.001) (derived from repeated-measures ANOVA and adjusted using Bonferroni correction).

**Figure 3:**
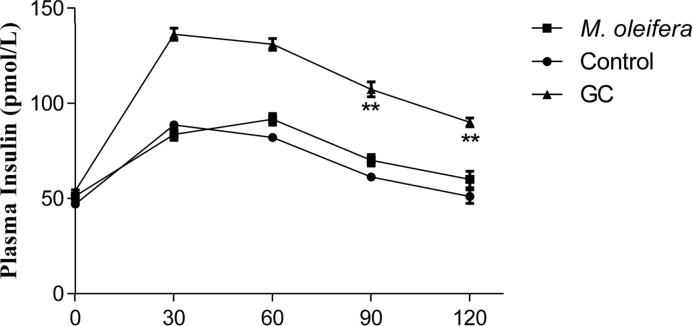
Effects of ethanol extract of *M. oleifera* on plasma insulin level in Type 2 diabetic rats Values are means ± S.E.M represented by vertical bars (*n*=8). Rats were given ethanol extract of *M. oleifera* (500 mg/kg) or Glibenclamide (GC) (0.5 mg/kg) or only water (control) by oral administration. Mean values marked with an asterisk (*) were significantly different from those of respective control rats (**P <0.001) (derived from repeated-measures ANOVA and adjusted using Bonferroni correction).

Oral administration of *M. oleifera*, at 500 mg/kg, altered the hyperglycaemic condition of fasted, Type 2 diabetic rats after 30 min (Figure 1). The extract also showed significant effect on glucose tolerance at 90 min and 120 min but there was no significant increase in plasma insulin level after an acute glucose insult (Figures 2 and 3).

### Effect of *M. oleifera* on serum glucose after sucrose load

**Figure 4:**
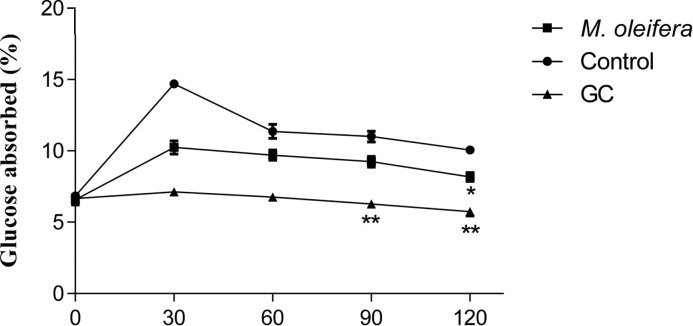
Effects of ethanol extract of *M. oleifera* on serum glucose after the sucrose load in Type 2 diabetic rats Rats were fasted for 20 h and administered orally with a sucrose solution (2.5 g/kg body weight) with or without ethanol extract of *M. oleifera* (500 mg/kg body weight) or Glibenclamide (GC) (0.5 mg/kg) or only water (control). Means ± S.E.M. represented by vertical bars (*n=*8). Mean values marked with an asterisk (*) were significantly different from those of respective control rats (^*^P <0.05, **P<0.001) (derived from repeated-measures ANOVA and adjusted using Bonferroni correction).

*M. oleifera* showed a significant (*P*<0.05) suppression of serum glucose level after 60 min compared with control, where peak serum glucose was observed after administration of sucrose load and 500 mg/kg dose of *M. oleifera* maintained this trend of suppression of glucose level at 120 min too (Figure 4).

### Effect of *M. oleifera* on intestinal glucose absorption

**Figure 5:**
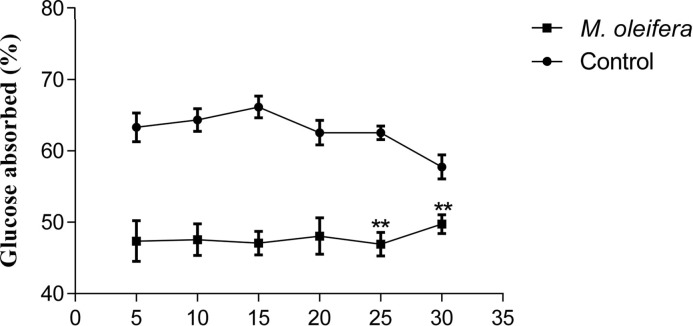
Effects of ethanol extract of *M. oleifera* on intestinal glucose absorption in Type 2 diabetic rats Rats were fasted for 36 h and the intestine was perfused with glucose (54 g/l) with (treated group) or without (control group) ethanol extract of *M. oleifera* (10 mg/ml each subject received 15 ml of perfusion). Values are means ± S.E.M represented by vertical bars (*n=*8). Mean values marked with an asterisk (*) were significantly different from those of respective control rats (^**^P <0.001) (derived from repeated-measures ANOVA and adjusted using Bonferroni correction).

Five hundred milligrams per kilogram doses of *M. oleifera* extract, when perfusated with glucose, showed significant (*P*<0.05) reduction in the percentage of glucose absorption during most of the perfusion period (Figure 5).

### Effect of *M. oleifera* on unabsorbed sucrose content in the GI tract

**Figure 6:**
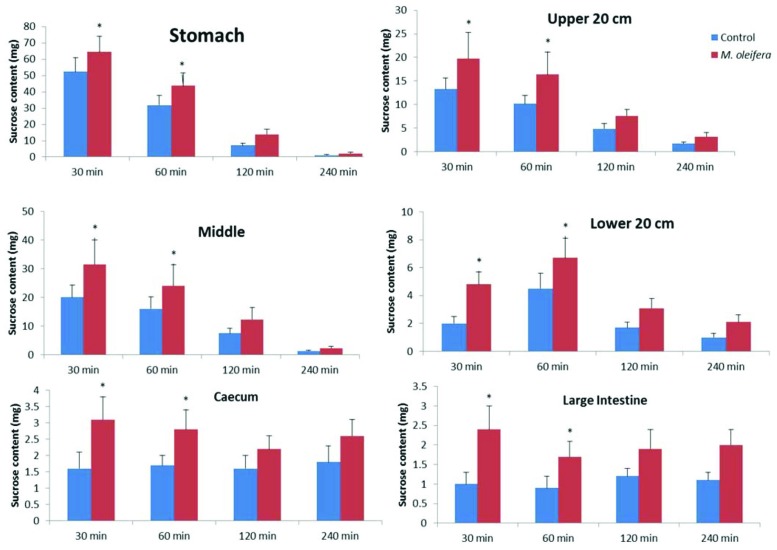
Effects of ethanol extract of *M. oleifera* on GI tract sucrose content after oral sucrose loading in Type 2 diabetic rats Rats were fasted for 20 h before the oral administration of a sucrose solution (2.5 g/kg body weight) with (treated group) or without (control group) ethanol extract of *M. oleifera* (500 mg/kg body weight). Values are means ± S.E.M represented by vertical bars (*n=* 8). Mean values marked with an asterisk (*) were significantly different from those of respective control rats (^*^P<0.05) (derived from repeated-measures ANOVA and adjusted using Bonferroni correction).

Upon oral administration of sucrose along with *M. oleifera* (500 mg/kg), significant amount of unabsorbed sucrose remained in the stomach, upper, middle and lower intestines at 30 min and 60 min. This amount of residual sucrose remained significant in caecum and large intestine till 4 h (*P*<0.05; Figure 6).

### Effect of *M. oleifera* on gut motility

**Figure 7:**
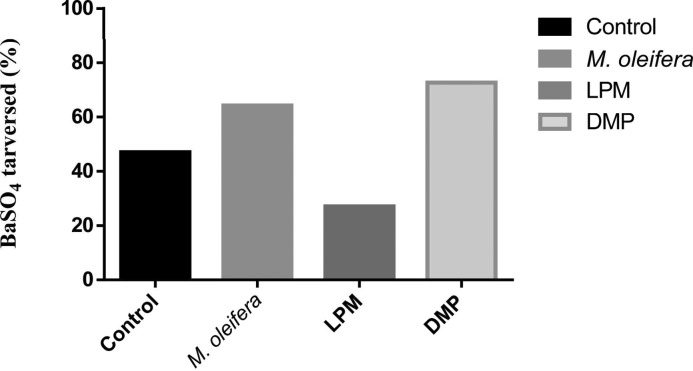
Effects of ethanol extract of *M. oleifera* on GI tract motility (by BaSO_4_ traversed).

*M. oleifera* extract increased the GI motility significantly (*P*<0.05) at 500 mg/kg dose in Figure 7.

Rats were fasted for 20 h before the oral administration of ethanol extract of *M. oleifera* or water (control). BaSO_4_ was administered at 60 min. Motility was measured over the following 15 min. Loperamide (LPM) (5 mg/kg) and domperidone (DMP) (10 mg/kg) were used as reference controls for GI motility test (*n*=12). Mean values and significance (*P*<0.05) were derived from repeated-measures ANOVA and adjusted using Bonferroni correction.

### Effect of *M. oleifera* powder on *in vitro* GDRI

*M. oleifera* powder reduced the amount of glucose present in the dialysate. GDRI was 36.97% and 46.20% at 30 and 60 min respectively (*P*<0.05; Table 1).

**Table 1:** Retarding effect of insoluble fibre of *M. oleifera* on the glucose movement (GDRI)

Treatment	Dialysis for 30 min		Dialysis for 60 min	
	Glucose in dialysate (mmol/l)	GDRI (%)	Glucose in dialysate (mmol/l)	GDRI (%)
*M. oleifera*	0.75 ± 0.18*	36.97	0.92 ± 0.12*	46.20
CM-cellulose 1000 mg	0.62 ± 0.11*	42.86	0.83 ± 0.07*	51.46
Control	1.19 ± 0.21	0	1.71 ± 0.14	0

Data are presented as means ± SD (*n*=3). Glucose dialysis retardation index = control (100%) – fiber (% of control value). Mean values marked with an asterisk (*) were significantly different from those of respective control groups (*P<0.05) (derived from repeated-measures ANOVA and adjusted using Bonferroni correction).

Data are presented as means ± S.E.M (*n*=4). GDRI = control (100%) – fibre (% of control value). Mean values marked with an asterisk (*) were significantly different from those of respective control groups (^*^P<0.05) (derived from repeated-measures ANOVA and adjusted using Bonferroni correction).

### Effect of *M. oleifera* powder on α-amylase activity

**Figure 8:**
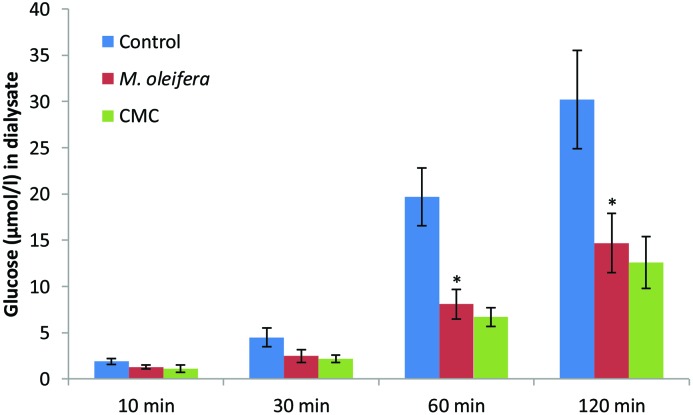
Effect of *M. oleifera* powder on α-amylase activity Data are presented as means ± S.E.M ( n= 4). Values are represent the glucose concentration (μmol) in dialysate. Mean values marked with an asterisk (*) were significantly different from those of respective control rats (^*^P<0.05) (derived from repeated-measures ANOVA and adjusted using Bonferroni correction).

The effect of *M. oleifera* powder on starch digestibility was determined by the alteration in the glucose concentration in the dialysate with time. There was significant change, compared with control, in the glucose content at 60 min and 120 min (*P*<0.05; Figure 8).

### Effect of *M. oleifera* powder on *in vitro* glucose-adsorption capacity

**Figure 9:**
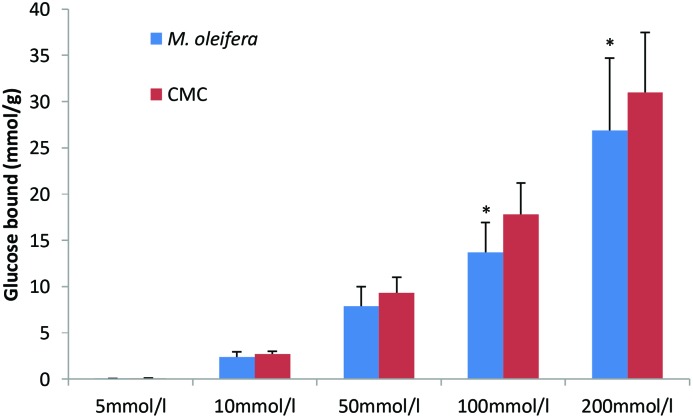
Effect of *M. oleifera* powder on *in vitro* glucose-adsorption capacity Data are presented as means ± S.E.M (*n*= 4). Values are represent the millimoles of glucose bound by each gram of *M. oleifera* powder at different glucose concentration (5–200 mmol/l). Glucose bound = (glucose concentration of original solution - concentration when the adsorption reached equilibrium) × volume of solution ÷ weight of dietary fiber. Mean values marked with an asterisk (*) were significantly different from those of respective control rats (^*^P<0.05) (derived from repeated-measures ANOVA and adjusted using Bonferroni correction).

*M. oleifera* powder depicted the capacity of glucose adsorption in the presence of different levels of glucose in the solution. This activity of glucose adsorption was found to continue from higher level of glucose to low level of glucose present in the solution (Figure 9).

## Discussion

Streptozotocin used in the present study to induce Type 2 diabetes in adult animals that causes DNA damage and generates superoxide radicals to destroy the β cells [26]. We have used Glibenclamide (GC) in this experiment as a reference drug, which is a synthetic hypoglycaemic agent and has been used as an anti-diabetic drug in Type 2 diabetic patients since 1973 and is still being used [27]. This drug acts by stimulation of insulin release [28]. Only one oral dose of 500 mg/kg BW of *M. oleifera* ethanolic leaf extract was administered for evaluation of anti-hyperglycaemic properties by increasing blood glucose tolerance in the normal rat, which was less potent than those of STZ-induced diabetic rat. *M. oleifera* leaves contain many powerful antioxidant phytochemicals, especially quercetin and kaempferol [29]. There are many reports about hypoglycaemic activities of kaempferol derivatives from many medicinal plants such as *Sterculia rupestris* [30] and *Equisetum myriochaetum* [31]. Furthermore, they also improved chronic hyperglycaemia impaired pancreatic β cells viability and insulin secretion *in vitro* [32]. Quercetin, a strong antioxidant flavonoid revealed a protective effect against STZ-induced diabetes in rats by intraperitoneal injection of quercetin 15 mg/kg BW for 3 days prior to STZ administration [33] and protected an insulin secreting cell line (INS-1) against oxidative damage [34,35]. It also exhibited hypoglycaemic properties in diabetic rats [36]. Vessal et al. [37] reported that quercetin significantly increased hepatic glucokinase activities as an insulin-like effect.

Hyperglycaemia causes cellular damage that hinders homoeostasis of internal glucose concentration, which results in acutely altered cellular metabolism and long-term changes in cellular macromolecular content [38–40]. Postprandial glucose spike causes perturbation in endothelial cell function, [41,42] and increases the risk of blood coagulation [42]. Hyperglycaemic states also increase products of glycosylation, which has a significant influence on the development of diabetes-induced vascular disease [43]. Therefore, management of hyperglycaemic states is an important method of diabetes control. There are some basic pathways used in anti-diabetic drugs that include enhanced insulin secretion, enhanced sensitivity to insulin, improved peripheral glucose utilization, inhibition of glucose absorption and inhibition of carbohydrate digestion [44]. *M. oleifera* leaves have shown promising glucose-lowering effect in the present study with chemically induced hyperglycaemic rats. However, there are no reports that have been issued to reveal the tissue level mechanism of action of *M. oleifera*. In our present study, we have employed different techniques, which suggest one or more of the aforementioned modes of action.

Different studies have proved that blood glucose level in the upper normal range is a probable risk factor for cardiovascular disease, a common condition in case of chronic Type 2 diabetic patients [45]. In our study, fasting blood glucose in Type 2 diabetic rats in treatment group was changed significantly (p<0.05) and it was highly significant when compared to standard (Glibenclamide). In glucose tolerance test, the peak glucose concentration after glucose induction whereas *M. oleifera* treated group (500 mg/kg) significantly (p<0.05) declined the glucose absorption after 60 min compared to control group.

To further ascertain this, we measured the plasma insulin level of the test animals and found no significant increase in insulin secretion on *M. oleifera* administration. Therefore, increased insulin secretion from pancreatic β cell can be ruled out as possible mechanism of action.

An *in situ* intestinal perfusion of the GI tract shows marked reduction in glucose absorption. In BaSO_4_ GI motility assay, intestinal motility was found to be significantly higher. Six Segment test showed significantly higher amount of unabsorbed sucrose in stomach, upper, middle and lower intestines in *M. oleifera*-administered groups. The last three parts of GI tract are most important for absorption of nutrients including sugars [46]. Highly unabsorbed sucrose content in the GI tract indicates that sucrose digestion has been reduced. Thus, a significantly higher concentration of sucrose has reached to the large intestine and caecum, which eventually remains unabsorbed and egested with faeces. An *in vitro* study was conducted to evaluate fibre-binding capacity of the crude extract, no substantial effect was found that clearly demonstrates no dietary fibres are available in *M. oleifera*. Glucose is carried by specific transporter proteins [47]. Bound glucose is probably incapable of fitting the active site of these transporter proteins. This finding validates our initial findings in the gut perfusion experiments, which too showed no hindrance in glucose absorption. This is now fully understood that there is no glucose-fibre binding capacity of the crude extract.

*M. oleifera* in the α-amylase activity assay showed promising decrease in the catabolism of starch. As complex carbohydrates and disaccharides need to be broken down into simpler monosaccharides [48] prior to absorption, any inhibition of this catabolic process would retard sugar absorption, which in turn lowers glycaemic peak. However, the precise mechanism of this inhibitory action remains to be studied.

## Conclusions

The present study showed that ethanolic leaves extract of *M. oleifera* possessed hypoglycaemic and anti-hyperglycaemic properties in chemically induced Type 2 diabetic rats, which suggest the presence of biologically active components that may be worth further investigation and elucidation. Studies confirm the previous claims regarding anti-hyperglycaemic action of *M. oleifera*. Additionally, we have elucidated that *M. oleifera* is capable of inhibiting absorption of glucose by inhibition of α-amylase. Therefore, its traditional use, as mentioned above, is justified and a primary mechanism of action is now known that was unrevealed until the present study.

## Abbreviations

ACB: acarbose
GC: glibenclamide
GDRI: glucose dialysis retardation index
GI: gastrointestinal
STZ: streptozotocin
BW: body weight
GOD-PAP: enzymatic colorimetric method for glucose determination
